# Intersectoral Strategies for Type 2 Diabetes Prevention and Management in Emerging Countries: A Narrative Review of Food Sovereignty, Digital Health, and Syndemic Dynamics

**DOI:** 10.3390/ijerph22101572

**Published:** 2025-10-15

**Authors:** Tatiana Palotta Minari

**Affiliations:** Department of Bioscience, Federal University of São Paulo (UNIFESP), Santos 11015-020, SP, Brazil; tatiana.minari@unifesp.br or tatianaminari@gmail.com

**Keywords:** syndemics, Type 2 diabetes, food insecurity, digital health equity, intersectoral governance, agroecology, health systems innovation, global south

## Abstract

Background: Type 2 diabetes (T2D) is no longer a standalone clinical condition—it has become a syndemic shaped by food insecurity, social inequality, and digital marginalization in emerging economies. This convergence calls for a reimagining of public health through intersectoral, digitally enabled, and culturally grounded approaches. This study explores how intersectoral strategies, supported by digital innovation and rooted in food sovereignty, can help prevent and manage T2D in emerging countries. Methods: A narrative review was conducted using the PubMed, Scopus, and Web of Science databases. Studies published between 2014 and 2025 were included if they addressed T2D and food security in emerging contexts, focusing on intersectoral or multisectoral strategies. Studies on T1D, non-human subjects, and high-income settings were excluded from the study. Thirty-nine studies were critically synthesized. Results: Food insecurity acts as both a biological stressor and a sociopolitical condition that worsens poor glycemic control. Promising but underutilized intersectoral strategies include agroecological food systems, school-based nutrition programs, and community health worker networks. Digital tools, such as AI-driven diagnostics, blockchain food traceability, and mobile health platforms, offer scalable solutions but face challenges in infrastructure, digital literacy, and ethical governance. Conclusions: A digitally inclusive, ethically reflexive intersectoral paradigm is needed that recognizes food and digital access as human rights.

## 1. Introduction

Type 2 diabetes (T2D) has become a global syndemic—a complex crisis where biological, social, environmental, and technological factors interact to increase the disease burden, especially in emerging economies [1,2]. In these settings, rapid urbanization, shifts in nutrition toward ultra-processed foods, and systemic inequalities combine with digital exclusion and ecological degradation [2,3]. The condition disproportionately impacts marginalized populations, including racialized communities, low-income households, and women, who often navigate environments marked by food insecurity, fragmented health systems, and limited access to digital infrastructure [4,5].

Food security and food sovereignty are often viewed as separate approaches to tackling global nutrition and fairness. Food security focuses on the availability, access, and use of enough, safe, and nutritious food to satisfy dietary needs and preferences for a healthy and active life. On the other hand, food sovereignty highlights the right of communities to shape their own food systems, emphasizing local production, culturally suitable practices, and democratic influence over agricultural policies [4,5].

Food insecurity, in this context, is not just a nutritional problem—it is a sign of structural violence and political neglect [6]. It shows the breakdown of traditional food systems, land dispossession, global trade inequalities, and the commercialization of nutrition. People facing food insecurity experience ongoing stress, poor glycemic control, and higher risks of complications like cardiovascular disease and diabetic ketoacidosis [7]. The mental and emotional effects—such as anxiety, depression, and social isolation—further hinder self-care and widen health disparities [8]. These issues are worsened by the fact that food insecurity often goes unnoticed in clinical settings, where biomedical models tend to overlook the social, political, and emotional factors of chronic illness [6].

Intersectorality has emerged as a strategic framework to address these complexities. In the context of public health systems, intersectorality refers to the intentional and coordinated integration of policies, actions, and resources across different sectors—such as health, education, agriculture, and social protection—to address complex health determinants holistically [5,6,7]. Unlike multisectoral collaboration, which often involves parallel efforts by various sectors working independently toward a shared goal, intersectorality implies deeper institutional alignment, joint planning, and shared accountability [6,7]. For example, a multisectoral initiative might involve the health and education sectors, each running separate campaigns on child nutrition. In contrast, an intersectoral approach would entail these sectors co-designing a unified strategy, pooling resources, and implementing joint interventions that address both nutritional education and access to healthy food in schools [8].

Promising initiatives, such as Brazil’s National School Feeding Program (PNAE), agroecological cooperatives in Colombia, and vegetable prescription models in the U.S., demonstrate the potential of multisectoral approaches to improve dietary quality, community resilience, and chronic disease outcomes [9,10,11,12,13,14,15]. However, these interventions remain fragmented, underfunded, and disconnected from the broader systems of care. Most lack integration with digital infrastructure, cultural epistemologies, and mechanisms for sustained community engagement [12,13,14,15].

The digital era has introduced both opportunities and risks [16]. Mobile health applications, artificial intelligence (AI)-powered diagnostics, blockchain for food traceability, and ecological calendars for climate adaptation offer new tools for chronic disease management and food system transparency [17]. However, these innovations are unevenly distributed and often inaccessible to the populations most affected by T2D. Digital literacy gaps, algorithmic bias, and data colonialism threaten to reproduce existing inequities under the guise of technological progress [18]. Without inclusive design, ethical safeguards, and culturally relevant implementation, digital health solutions risk becoming an additional layer of exclusion [19].

Despite the growing interest in intersectoral approaches and digital health innovations, the literature remains fragmented and conceptually limited. Few studies have examined the intersection of food insecurity, chronic disease, and digital transformation through a critical, equity-focused lens [20,21,22,23,24,25]. Most research isolates sectors—health, education, and agriculture—without exploring their systemic interdependencies or the role of digital tools in bridging these divides [26,27,28,29,30,31]. Moreover, there is a lack of frameworks that integrate food sovereignty, digital justice, and syndemic theory into chronic disease prevention and management [32,33,34,35,36,37,38,39,40].

This critical narrative review seeks to address a significant gap in the literature by examining how intersectoral strategies, enhanced by digital innovation and grounded in the principles of food sovereignty, can be mobilized to prevent and manage T2D in emerging countries, with emphasis on digital innovations such as mobile health platforms, AI-driven diagnostics, and blockchain-based food traceability systems. By exploring the complex interplay between chronic illness, structural inequality, and technological transformation, the study proposes a roadmap for building integrated, equitable, and culturally responsive public health systems.

## 2. Materials and Methods

The narrative review format was selected for its ability to integrate diverse forms of evidence—empirical, theoretical, and policy-oriented—across disciplines and sectors. This approach is particularly suited to exploring complex intersections such as syndemics, food sovereignty, and digital health equity, which are often marginalized in conventional biomedical research.

For this study, “emerging countries” were defined according to the World Bank classification of lower-middle and upper-middle income economies, with additional consideration given to nations undergoing rapid digital transformation and facing structural challenges in public health and food systems. This definition was applied consistently throughout the study selection process to ensure contextual relevance.

A comprehensive literature search was conducted across four major databases—PubMed, Scopus, and Web of Science—using a structured strategy based on the PCC framework (population, concept, context). The search terms included the following: Population (“Type 2 diabetes” OR “T2D”), Concept (“Food insecurity” OR “Food security” OR “Nutrition access” AND “Intersectoral collaboration” OR “Multisectoral strategies” OR “Health systems” OR “Social protection” OR “Education” OR “Agriculture” OR “Digital health”), and Context (“Emerging countries” OR “Low- and middle-income countries” OR “LMICs” or specific country names based on World Bank classification). Boolean operators (“AND,” “OR”) were applied to refine the search strings across all databases. Separate search strings were tailored for each database. In PubMed, controlled vocabulary (MeSH terms) such as “Diabetes Mellitus, Type 2” [MeSH] and “Food Security” [MeSH] were combined with free-text terms to optimize retrieval. Scopus and Web of Science searches used equivalent keyword combinations, adjusted for each database’s syntax. The search strategy based on the PCC framework is summarized in Table 1.

Studies were considered eligible if they were published between 2014 and 2025, addressed T2D and food security within the context of emerging economies, and engaged intersectoral or multisectoral strategies involving health, education, agriculture, or social protection. Articles written in English were included if they provided empirical data, policy analyses, or case-based insights. Gray literature—including policy briefs, reports, and government documents—was also considered if they met the inclusion criteria and provided relevant insights into intersectoral strategies. Exclusion criteria ruled out studies focused exclusively on Type 1 diabetes or non-human subjects, as well as those conducted in high-income countries or lacking relevance to intersectoral or digital health dimensions.

**Figure 1:**
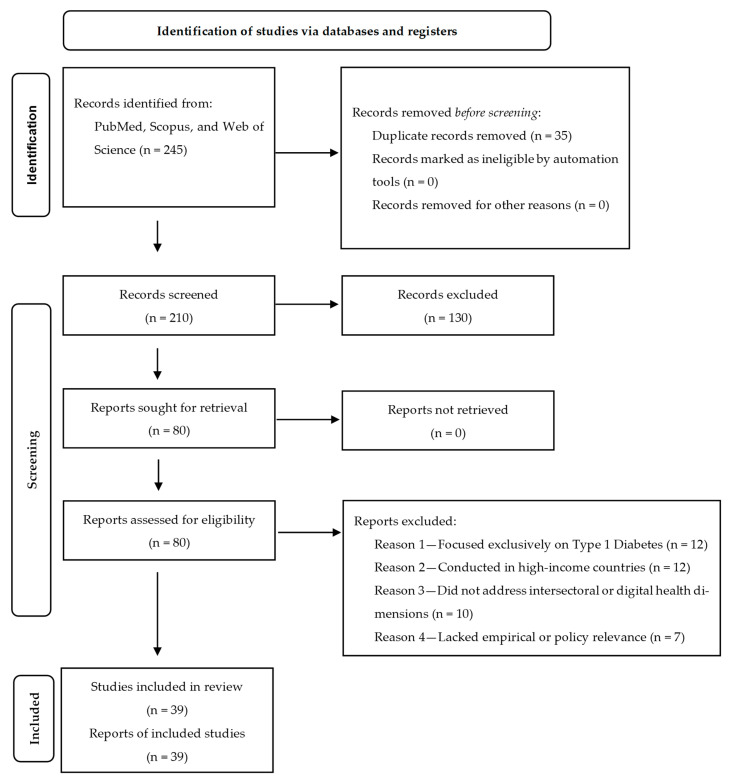
PRISMA flow diagram of study selection process. Visual representation of the identification, screening, eligibility assessment, and inclusion of studies in the narrative review. The diagram outlines the number of records retrieved from databases, the number of duplicates removed, the articles excluded at each stage, and the final set of studies included in the synthesis. Adapted from PRISMA 2020 flow diagram, available at http://www.prisma-statement.org (20 August 2025).

From an initial pool of 245 records, 80 articles were selected for full-text screening. After applying the inclusion and exclusion criteria, 39 studies were retained for the final synthesis. The study selection process is visually summarized in a PRISMA flow diagram (Figure 1), which outlines each stage of the screening and eligibility assessment.

Data extraction was performed manually using a standardized form developed on the Rayyan^®^ (Doha, Qatar). This form was designed to systematically capture key variables across the selected studies, including the study design, population characteristics, type of intersectoral strategy, integration of digital health tools, and outcomes related to glycemic control, food access, and community resilience. Additional fields were included to assess the scalability, cultural relevance, and policy implications of each intervention, ensuring consistency across sources and supporting a thematic synthesis aligned with the review’s objectives.

Screening and selection were conducted by a single reviewer using predefined inclusion and exclusion criteria. To enhance consistency and reduce bias, the selection process followed a structured protocol, and ambiguous cases were re-evaluated based on their alignment with the study objectives and eligibility parameters.

A narrative risk of bias assessment was conducted using the JBI Critical Appraisal Checklist for Qualitative Research^®^ (Adelaide, Australia). Methodological limitations were noted and considered in the interpretation of the findings.

The narrative synthesis allowed for the juxtaposition of diverse approaches, highlighting both the convergences and gaps in the literature. The geographic scope included Latin America, sub-Saharan Africa, South Asia, and the Middle East—regions where food insecurity and chronic diseases intersect with systemic inequities and digital exclusion.

## 3. Results

### 3.1. Methodological Diversity and Scope

The studies reviewed encompass a wide range of methodological designs, including randomized clinical trials, meta-analyses, cross-sectional surveys, cohort studies, policy, international agreement, and strategic reviews, reflecting the multidimensional nature of the topic and the need for integrative, multisectoral approaches. Quantitative studies provided epidemiological data on health outcomes, such as odds ratios and prevalence rates, while qualitative and mixed-methods research offered insights into lived experiences, governance models, and cultural dynamics. This diversity supports a comprehensive understanding but requires clearer stratification to distinguish between evidentiary weight and methodological rigor.

### 3.2. Food Sovereignty as a Conceptual Lens

A prominent conceptual advancement in the literature is the articulation of food sovereignty as a framework that transcends conventional food security paradigms. Rather than focusing solely on access and availability, food sovereignty emphasizes autonomy, cultural relevance, and systemic justice. This lens has proven analytically robust in qualitative studies examining community-level responses to crises such as the COVID-19 pandemic, where local actors demonstrated resilience and adaptive governance in the face of institutional breakdowns. The indicators used to operationalize food sovereignty included local food production rates, community-led distribution initiatives, and participatory decision-making structures.

### 3.3. Digital Innovations and AI Applications

Technological innovation, particularly through the application of artificial intelligence, has emerged as a frontier in both clinical and systems-level interventions. Quantitative studies have employed AI to optimize insulin management, predict long-term diabetes risk, and model sustainable food systems. For example, predictive models achieved high accuracy (AUC = 0.89) [19], and insulin algorithms reduced hypoglycemic events by 27% in one study [18]. While these approaches demonstrate strong potential for scalability and precision, their implementation remains constrained by infrastructural limitations in low-resource settings. Moreover, the lack of longitudinal validation and equity-focused design raises concerns regarding generalizability and inclusivity, particularly for marginalized groups.

### 3.4. Epidemiological Evidence and Health Outcomes

From an epidemiological standpoint, the literature consistently establishes a correlation between food insecurity and adverse health outcomes. These include poor glycemic control, increased cardiovascular risk, and heightened vulnerability among adolescents, pregnant women, and racialized populations. Cross-sectional and cohort studies have reported that adolescents experiencing food insecurity had 2.3 times higher odds of poor glycemic control, and pregnant women faced a 1.8-fold increased risk of gestational hypertension. Racialized groups experienced food insecurity at a rate of 42%, compared to 18% among non-racialized populations [33]. These findings reinforce the imperative to integrate nutrition-sensitive strategies into primary healthcare, especially in regions marked by structural inequities and limited access to preventive services.

### 3.5. Political and Ethical Dimensions

The political and ethical dimensions of hunger have been critically examined in qualitative and theoretical studies, which frame food insecurity not merely as a nutritional deficit but as a systemic failure of public health governance. The recurring themes included poverty, social exclusion, and environmental degradation. This framing calls for a paradigm shift toward justice-oriented policies that address the root causes of food deprivation and advocate for structural reforms grounded in human rights, sustainability, and redistributive justice.

### 3.6. Clinical Interventions and Cultural Relevance

Clinical and intervention-based research has provided empirical evidence for the efficacy of targeted nutritional strategies in improving glycemic outcomes and mitigating cardiovascular risk. These interventions were most effective when they were culturally contextualized and integrated into broader public health initiatives. For example, a 12-week dietary program tailored to traditional practices among *Quilombola* communities led to a 1.2% reduction in HbA1c (*p* < 0.01), whereas community-based nutrition education in urban peripheries increased treatment adherence by 35% [25]. These outcomes underscore the importance of tailoring care models to local dietary practices, resource availability, and community engagement.

Table 2 presents a synthesis of these studies included in the narrative review. Each entry is categorized by core theme, source type, methodology, policy or programmatic relevance, critical insights, limitations, and future perspectives. This format provides a comprehensive overview of the key findings and strategic opportunities for advancing intersectoral responses to T2D and food security in emerging contexts.

## 4. Discussion

The synthesis of the selected literature reveals that food insecurity operates not only as a precursor to T2D but also as a compounding factor that undermines disease management and exacerbates health inequity [23]. In emerging countries, the cyclical relationship between poor nutrition, metabolic dysfunction, and socioeconomic vulnerability is intensified by systemic fragmentation across the health, education, and social protection sectors [24]. Individuals facing food insecurity often rely on ultra-processed foods that are high in sugar, sodium, and saturated fats—dietary patterns strongly associated with insulin resistance and poor glycemic control. These nutritional deficits are not merely behavioral choices but are structural outcomes shaped by poverty, market dynamics, and policy neglect [25].

Beyond its physiological consequences, food insecurity disrupts adherence to medical and nutritional guidance. Studies from Ethiopia, Brazil, and South Africa have demonstrated that patients experiencing food insecurity are less likely to follow dietary counseling, not due to a lack of knowledge, but because of economic constraints and cultural dissonance between clinical recommendations and lived realities [7,8,9,10,24]. This underscores the need for culturally tailored nutrition strategies that respect local food, economic conditions, and community agencies.

Primary healthcare systems in many emerging countries remain under-resourced, fragmented, and poorly integrated. Workforce shortages, limited diagnostic capacity, and vertical programming hinder the delivery of continuous and holistic diabetes care [24]. However, when reinforced by community health workers, multi-professional teams, and territorial governance, primary care can become a transformative platform for early detection, patient education, and follow-up [22,23,24]. Innovative models, such as “food as medicine” and vegetable prescription programs, illustrate how health systems can transcend clinical boundaries by directly addressing food access and nutritional support. These interventions not only improve glycemic control but also foster trust, social cohesion, and community engagement [25].

Schools represent a strategic yet underutilized arena for preventing chronic diseases. Programs like Brazil’s PNAE and similar initiatives in Colombia and South Africa [24,25] show that school feeding can enhance dietary quality and reduce the risk of noncommunicable diseases [25]. However, most educational systems lack structured health curricula, and teachers are seldom equipped to deliver content on nutrition, physical activity, or diabetes prevention. Integrating health education into school programs, alongside training for educators and community involvement, could cultivate lifelong habits and empower children as agents of change within their families and territories [9,24].

Social protection mechanisms, such as conditional cash transfers, food subsidies, and nutritional assistance, play a critical role in mitigating the economic determinants of poor health. However, their effectiveness depends on context-sensitive design and integration with health systems [25]. Evidence from high-income countries such as the United States shows mixed outcomes, with programs such as SNAP yielding variable effects on glycemic control. In emerging contexts, linking food support with health monitoring, digital outreach, and community participation can amplify impact—especially when aligned with local dietary norms and infrastructure realities [26].

A particularly underexplored dimension in the literature is the role of digital technologies in reshaping the landscape of food security and chronic disease care [27]. The digital era has introduced new tools, including mobile health applications, AI-powered diagnostics, blockchain for food traceability, and ecological calendars for climate adaptation, which hold promise for enhancing transparency, personalization, and scalability [17]. For instance, AI algorithms can predict glycemic fluctuations based on dietary inputs, behavioral patterns, and biometric data, thereby enabling proactive interventions. Blockchain systems can ensure the integrity of food supply chains, reduce fraud, and support local producers through transparent transactions [18]. Mobile platforms can deliver culturally relevant nutrition education, facilitate remote consultations, and monitor treatment adherence in real-time [19].

Empirical applications of these technologies in resource-constrained settings are emerging. In India, AI-powered mobile platforms have been used to monitor dietary habits and predict glycemic risk among rural populations, enabling targeted interventions for diabetes prevention in rural areas [18,35]. In sub-Saharan Africa, blockchain systems have been piloted to trace food supply chains and support smallholder farmers by improving market access and reducing postharvest losses. In Brazil, mobile health apps have facilitated remote consultations and nutrition education in underserved urban peripheries, improving treatment adherence and dietary outcomes in patients with chronic conditions [17]. These examples illustrate the potential of digital tools to enhance health equity and food system resilience when adapted to local contexts [40].

However, these innovations are not neutral in their effects. They are embedded within power structures that can reproduce or exacerbate inequitable conditions. The digital divide—manifested as unequal access to devices, connectivity, and digital literacy—limits the reach of these tools among the populations most affected by TD2 [17]. Algorithmic bias, data colonialism, and surveillance capitalism pose ethical challenges that must be addressed through inclusive design, participatory governance, and the establishment of regulatory safeguards. Without critical engagement, digital health risks become another layer of exclusion, reinforcing the invisibility of marginalized communities in data-driven systems [17,18,19].

To address these ethical challenges, it is essential to move beyond critique toward structured governance. The World Health Organization (WHO) has proposed guidelines for digital health ethics that emphasize transparency, accountability, inclusiveness, and equity in the design and deployment of digital technology [40]. These principles advocate participatory governance models that actively involve affected communities in decision-making processes, promote algorithmic transparency, and safeguard data sovereignty. In the context of emerging countries, such frameworks must be adapted to local realities by integrating ethical oversight into digital health policies, investing in community-based digital literacy, and ensuring that innovation aligns with public health equity goals. Without such governance, digital health risks reinforce existing asymmetries rather than resolving them [17,18,19].

The concept of digital food deserts—areas where access to online food resources, delivery services, and nutritional information is limited—adds another layer to this discussion. In many urban peripheries and rural regions, the digital infrastructure is insufficient to support telehealth, e-commerce, or digital education [17]. This not only affects food access but also undermines the potential of digital health interventions. Addressing these gaps requires investment in digital public goods, community-based digital literacy programs, and policies that prioritize connectivity as a determinant of health [18].

The psychosocial burden of food insecurity and chronic illnesses is profound yet often overlooked. Individuals navigating economic hardship and dietary limitations frequently experience anxiety, depression, and social isolation, which exacerbate diabetes outcomes and hinder self-management [33,34,35,36]. Studies conducted in Ethiopia, Brazil, and South Africa have documented high prevalence rates of psychological distress among patients with T2D living in food-insecure households, with depression rates ranging from 30% to 50% depending on the context and screening tools used [7,8,9,10,24]. In Brazil, a cross-sectional study found that food-insecure individuals with diabetes were significantly more likely to report symptoms of anxiety and low treatment adherence, even when controlling for socioeconomic status [24]. Similarly, qualitative research in South Africa revealed that stigma, emotional fatigue, and lack of social support are major barriers to dietary compliance and glycemic control [10].

To address these challenges, validated screening tools, such as the Generalized Anxiety Disorder-7 (GAD-7) [41] and Patient Health Questionnaire-9 (PHQ-9) [42], should be considered for integration into diabetes care pathways. These instruments enable the early identification of mental health needs and facilitate timely referrals to support services. Integrated mental healthcare, peer support networks, and trauma-informed models are critical for fostering emotional resilience and community healing. Intersectoral strategies must incorporate these dimensions, recognizing that chronic disease is not only a biomedical condition but also a deeply social and emotional experience. Future research should prioritize longitudinal studies that examine the bidirectional relationship between food insecurity and mental health among individuals with diabetes, particularly in low-resource settings [33,34,35,36].

Global frameworks such as the 2030 Agenda for Sustainable Development offer strategic alignment for addressing the interconnected challenges of food insecurity and chronic diseases. Goals such as SDG 2 (Zero Hunger), SDG 3 (Good Health and Well-Being), and SDG 10 (Reduced Inequalities) are directly relevant to this discussion. However, translating these goals into actionable policies requires more than technical solutions; it demands political will, cross-sectoral collaboration, and participatory governance. The COVID-19 pandemic further exposed systemic vulnerabilities, amplifying food insecurity and disrupting chronic disease care, especially among marginalized communities [28]. This underscores the urgency of resilient health systems and inclusive social protection. Without inclusive planning and accountability mechanisms, global agendas risk remaining aspirational rather than transformative [28,29,30].

To translate these findings into actionable strategies, policies, and research efforts must be grounded in concrete governance and financing models that support intersectorality. Brazil’s *Food Acquisition Program* (FAP) demonstrates how public procurement can simultaneously strengthen local agriculture and improve food access for vulnerable communities [24,25]. Rwanda’s Community-Based Health Insurance (CBHI) offers a participatory financing model that links health services to agricultural cooperatives and expands access to chronic care. India’s Ayushman Bharat Digital Mission provides a scalable digital infrastructure that connects health records with nutrition and welfare programs, thereby enabling coordinated action across sectors. These models illustrate how intersectorality can be operationalized through integrated governance and hybrid financing mechanisms, such as results-based funding, pooled donor models, and social impact bonds. Future research should evaluate these approaches using equity-focused metrics and implementation science to ensure their sustainability and adaptability in diverse sociopolitical contexts [21,22,23].

**Figure 2:**
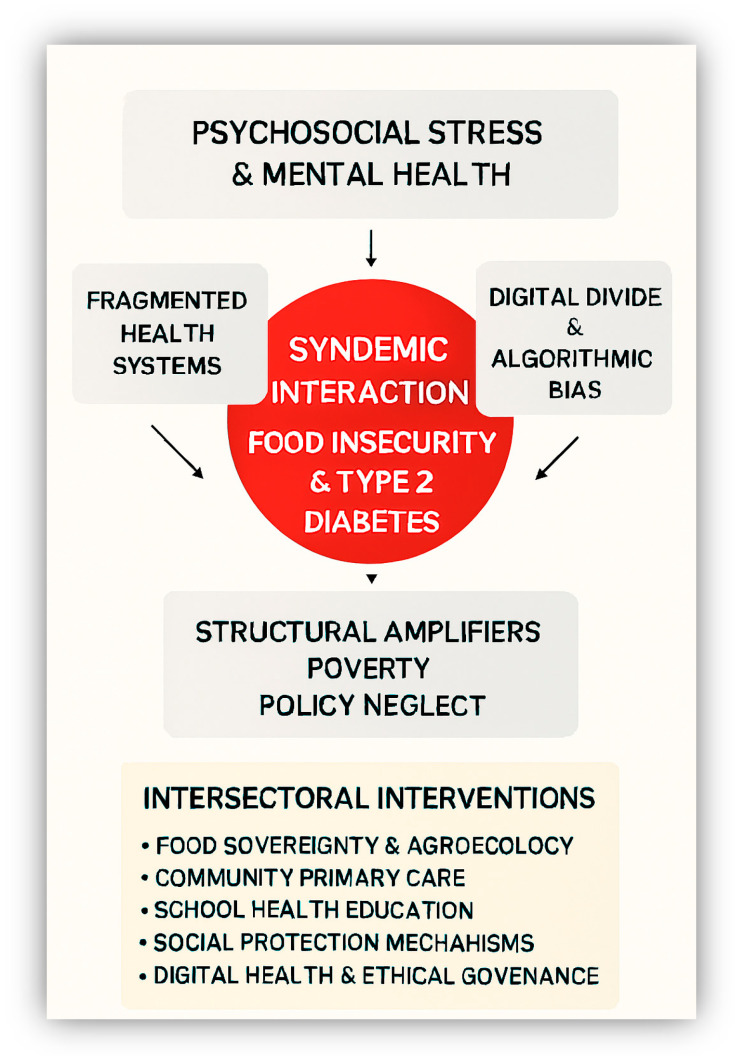
Syndemic Interaction Between Food Insecurity and T2D. This diagram depicts the multifaceted interplay of psychosocial, structural, and technological determinants that contribute to the syndemic relationship between food insecurity and T2D. Encircling the central interaction are key amplifying factors: psychosocial stress and mental health challenges, fragmented health systems, the digital divide coupled with algorithmic bias, and broader structural drivers such as poverty and policy neglect. The lower section presents a set of intersectoral interventions designed to address and mitigate these interconnected issues, including food sovereignty and agroecological practices, community-based primary care, school-based health education, social protection mechanisms, ethical digital health governance, and integrated mental health support.

This conceptual framework (Figure 2) synthesizes the syndemic interactions between food insecurity and T2D, highlighting the structural amplifiers and multidimensional factors that exacerbate the disease burden in emerging contexts. By mapping intersectoral intervention pathways, ranging from food sovereignty and school-based education to digital health and mental health integration, the figure underscores the need for coordinated, equity-driven responses. It also emphasizes enabling conditions such as participatory governance and policy coherence, which are essential for translating evidence into sustainable action. This visual model serves as a summary of a strategic guide for future research, policy design, and cross-sector collaboration.

The findings of this review resonate with global T2D prevention strategies, such as those promoted by the WHO [41] and American Diabetes Federation [37,38]. These frameworks emphasize early diagnosis, equitable access to care, and integration of nutrition into primary health systems. However, the studies reviewed here expand upon these models by incorporating food sovereignty as a central lens, highlighting autonomy, cultural relevance, and systemic justice as critical components of effective prevention. This conceptual advancement is particularly relevant in low-resource settings, where conventional approaches often fail to address the structural determinants of health [38]. Moreover, the inclusion of digital innovations, such as AI-based tools for insulin management and risk prediction, aligns with global trends toward precision medicine, while also raising important questions about equity and scalability [18,19].

These results have important implications for public health policies and practices. First, they suggested that food insecurity and T2D prevention must be addressed through multisectoral strategies that integrate health, agriculture, and social protection. Justice-oriented policies should prioritize culturally grounded nutritional interventions and support community-led food systems, especially in the context of institutional fragility [20]. The review also underscores the need to invest in digital infrastructure to enable the responsible use of AI in clinical and systems-level interventions. However, such technologies must be designed with inclusivity in mind to avoid reinforcing the existing disparities. Finally, emergency preparedness plans should incorporate sovereignty-based food-distribution models to enhance resilience and reduce dependence on external aid during crises [21].

Building on the epidemiological and clinical evidence reviewed, it is clear that targeted interventions are essential for high-risk groups, such as adolescents, pregnant women, and racialized populations. For adolescents, school-based nutrition programs combined with digital monitoring tools can support early prevention and behavioral changes. Pregnant women would benefit from prenatal nutrition counseling integrated into maternal health services with culturally adapted dietary guidance. In racialized and low-income communities, community gardens, mobile health units, and culturally relevant meal plans can foster autonomy and improve glycemic outcomes in people with diabetes. These interventions should be co-designed with local stakeholders to ensure their relevance, sustainability, and community ownership [11,33].

### 4.1. Limitations

Although this narrative review offers a transdisciplinary synthesis of intersectoral strategies and digital innovations in the context of T2D and food insecurity, several limitations must be acknowledged. First, the methodological approach does not support statistical generalization or meta-analytic precision. Therefore, the findings should be interpreted as indicative rather than definitive, serving as a conceptual foundation for future empirical investigations rather than conclusive evidence.

Second, many of the included studies lacked longitudinal data, rigorous impact assessments, or comparative analyses, which restricts the ability to evaluate the long-term effectiveness and scalability of the intersectoral interventions. The digital health literature in emerging economies remains fragmented, with few studies grounded in equity frameworks, participatory designs, or culturally responsive metrics. Moreover, the integration of digital tools into chronic disease care is often discussed in theoretical terms, with insufficient attention to infrastructural limitations, algorithmic bias, and ethical governance.

Finally, despite the breadth of studies reviewed, the overall quality of the evidence presents notable constraints. A substantial portion of the literature relies on cross-sectional designs, limiting causal inference and the capacity to assess the sustained outcomes. The absence of longitudinal validation is particularly evident in studies involving AI applications and food sovereignty frameworks, both of which require extended observations to determine their effectiveness. Additionally, the lack of disaggregated data by race, sex, and socioeconomic status impedes the ability to evaluate differential impacts across vulnerable populations. These gaps underscore the need for future research to adopt more robust methodologies and equity-centered metrics that reflect the lived realities of those most affected by food insecurity and chronic diseases.

### 4.2. Strengths

This review presents several key strengths. It offers one of the first integrative and critically informed syntheses to examine the intersections between T2D, food insecurity, and digital innovation through the combined lenses of syndemic theory, food sovereignty, and intersectoral governance. To date, no previous review has approached this topic with such conceptual breadth and critical repertoire, bridging public health, digital ethics, and socio-political determinants of nutrition in emerging countries.

First, it advances a transdisciplinary synthesis that connects public health, digital innovation, and food sovereignty—fields that are rarely integrated into the same analytical framework. Second, it contributes to the conceptual development of syndemic theory by explicitly linking T2D to food and digital inequities in emerging contexts. Third, by adopting an intersectoral lens, the review highlights how agricultural, educational, and social protection policies can come together to address chronic diseases in a way that is structurally informed and justice-oriented. Additionally, the inclusion of digital health technologies—such as AI-driven diagnostics, blockchain-based food traceability, and mobile health platforms—broadens the discussion beyond traditional biomedical paradigms.

Finally, the review provides a critical, equity-oriented roadmap for future research and policy, demonstrating how culturally grounded, digitally inclusive, and sovereignty-based strategies can promote resilience and health justice in low- and middle-income countries. Overall, this study contributes not only by mapping evidence but by reframing how chronic disease, technology, and food policy are understood in the global health agenda.

### 4.3. Future Perspectives

This review paves the way for critical future research and policy development. There is an urgent need for longitudinal, mixed-methods studies that assess the impact of intersectoral strategies on glycaemic control and food access, mental health, and community resilience. These studies should incorporate intersectional analyses that account for race, gender, age, and geographical disparities. They should also be co-designed with affected communities to ensure relevance and ownership.

Future research must also explore the role of digital health tools in depth, moving beyond technological optimism to critically examining issues of access, literacy, surveillance, and data sovereignty. Evaluating the effectiveness of mobile health platforms, AI-driven diagnostics, and blockchain systems requires not only technical metrics but also ethical, cultural, and political lenses. Participatory technology design, community-based digital literacy programs, and inclusive data governance models should be central to this agenda.

Policy efforts should prioritize the development of integrated care models that combine nutrition, mental health, and chronic disease management within a cohesive, territorially grounded framework. School-based interventions hold untapped potential. They should be expanded to include trained educators, culturally relevant curricula, and family engagement strategies. Agroecological and food sovereignty systems must be scaled and supported through public investment, land reform, and climate adaptation policies. These models can be formally integrated into national diabetes prevention strategies by aligning agricultural subsidies with nutritional goals, incentivizing local food production, and embedding agroecological principles into dietary guidelines and public procurement programs. Pilot initiatives in countries such as Brazil and Ecuador demonstrate the feasibility of linking agroecology to chronic disease prevention through school feeding programs, urban gardens, and community-supported agriculture. Evaluating these interventions through health impact assessments and economic modeling can generate robust evidence to support broader policy adoption.

The role of health workers in local communities remains underdeveloped in many intersectoral strategies addressing food insecurity and chronic disease. Community health agents, nurses, and lay health workers are often the first point of contact for individuals navigating diabetes and related conditions, yet their potential is constrained by limited training, fragmented care models, and a lack of digital integration. Expanding task-shifting strategies, in which non-specialist workers are trained to deliver essential services, can improve coverage and reduce the system burden, particularly in underserved areas. Training models should include competencies in nutrition counseling, mental health screening, and digital literacy, enabling frontline workers to engage with mobile health platforms, remote monitoring tools, and electronic health records.

To enhance effectiveness, these professionals must be integrated into digital health ecosystems that support real-time data sharing, decision support, and teleconsultation. Embedding these tools within national health systems and ensuring interoperability with existing platforms can strengthen continuity of care and improve outcomes. Policy frameworks should prioritize investment in workforce development, digital infrastructure, and participatory training programs that reflect the cultural and contextual realities of each community.

Strengthening intersectoral data collection and monitoring is for evidence-based decision-making. This includes harmonizing indicators across sectors, investing in open-access data platforms, and fostering cross-disciplinary collaboration among researchers, practitioners, and policymakers. Participatory governance mechanisms—such as citizen councils, community audits, and digital feedback loops—can help ensure that interventions are contextually grounded, equitable, and responsive to the lived realities of those most affected.

Finally, the future of chronic disease prevention in emerging countries lies in the development of systems that are not only clinically effective but also socially just, ecologically sustainable, and digitally inclusive. This requires a shift from reactive care to proactive, community-driven resilience—anchored in food sovereignty, digital equity, and intersectoral solidarity. The literature reveals both promise and fragmentation. Intersectoral strategies have demonstrated potential to improve dietary quality, glycemic control, and community resilience, but they remain underutilized, poorly integrated, and disconnected from digital ecosystems. The digital era offers new tools for chronic disease care, yet these must be deployed with careful attention to equity, ethics, and cultural relevance. Food sovereignty provides a critical lens to reframe nutrition as a human right rather than a commodity, while mental health integration is essential for holistic care. The challenge ahead lies in building systems that are not only technically efficient but socially just, ecologically sustainable, and digitally inclusive.

## 5. Conclusions

Intersectoral collaboration stands as a foundational strategy for confronting the synergistic interplay between T2D and food insecurity in emerging countries, yet its transformative potential remains contingent upon a radical reconfiguration of public health systems—one that integrates culturally grounded care, territorial governance, and digitally inclusive innovation. The convergence of chronic disease with structural inequities, ecological degradation, and digital exclusion demands more than technical coordination; it requires a paradigm shift that recognizes food and digital access not merely as basic needs but as strategic rights central to health equity and community resilience. Embedding principles of food sovereignty, mental health integration, and ethical technology deployment into policy frameworks can catalyze a new generation of care models—ones that are adaptive, participatory, and ecologically sustainable. This vision calls for sustained investment in infrastructure, cross-sectoral capacity-building, and inclusive governance mechanisms that reflect the lived realities of marginalized populations. Only through such a multidimensional and justice-oriented approach can emerging countries build resilient systems capable of responding to the complex, interwoven crises of chronic illness, nutritional insecurity, and technological transformation.

## 6. Take a Home Message

- Food insecurity drives T2D in emerging countries, exacerbated by poverty, inequality, and digital exclusion.
- Cross-sector collaboration strengthens prevention and resilience when rooted in local governance and cultural relevance.
- AI, mobile health, and blockchain hold promise but require ethical application and digital inclusion.
- School meal programs, agroecology, and community health workers remain underutilized assets in chronic disease strategies.
- Food sovereignty frames nutrition as a human right, linked to sustainability, identity, and empowerment.
- Mental health and trauma-informed care are essential to address the psychosocial burden of food insecurity.
- Global goals such as the SDGs require local adaptation through inclusive, participatory planning.
- A paradigm shift is imperative: food and digital access must be recognized as human rights for health equity and chronic disease resilience.

## Figures and Tables

**Table 1:** Search Strategy Based on PCC Framework.

PCC Element	Keywords Used
Population	“Type 2 Diabetes” OR “T2D” OR “Diabetes Mellitus, Type 2” [MeSH]
Concept	(“Food insecurity” OR “Food security” OR “Nutrition access” OR “Food Security” [MeSH]) AND (“Intersectoral collaboration” OR “Multisectoral strategies” OR “Health systems” OR “Social protection” OR “Education” OR “Agriculture” OR “Digital health”)
Context	“Emerging countries” OR “Low- and middle-income countries” OR “LMICs” OR specific country names (World Bank classification)

Note: PubMed searches included MeSH terms combined with free-text keywords; equivalent combinations were applied in Scopus and Web of Science.

**Table 2:** Evidence Base for Intersectoral Approaches to Food Security and Diabetes Prevention.

Reference	Core Theme	Type of Source	Methodology Research Type	Policy/ Program Relevance	Critical Analysis	Limitations	Future Perspectives
Abbasi et al. (2025) [23]	Dietary diversity and cardiovascular risk in diabetes	Cross-sectional study	Nutritional risk assessment/ Quantitative	Connects food insecurity to broader health risks	Frames food diversity as protective	Single-country data	Replicate in diverse emerging contexts
Abdurahman et al. (2019) [10]	Food insecurity and T2D risk	Systematic review and meta-analysis	Quantitative synthesis	Positions food insecurity as a chronic disease determinant	Supports the syndemic framing of diabetes	High study heterogeneity	Strengthen longitudinal and equity-focused research
Alawode et al. (2023) [12]	SNAP participation and glycemic control	Cross-sectional study	NHANES data analysis/ Quantitative	Evaluates food assistance impact	Shows mixed outcomes for diabetes care	U.S.-based policy	Adapt for emerging country programs
Arigbede et al. (2023) [29]	Hunger and food sovereignty in the context of SDGs	Critical review	Strategic critique/ Qualitative	Urges evidence-based public health approaches	Connects food to global development goals	Abstract framework	Develop robust evaluation tools for food policies
Beltrán et al. (2022) [36]	Food insecurity and glycemic outcomes in T2D	Systematic review and meta-analysis	Quantitative synthesis of health outcomes	Links food insecurity to hyperglycemia and diabetes risk	Emphasizes structural determinants of health	Heterogeneity across studies and regions	Expand meta-analytic models to include digital health factors
Castro et al. (2022) [28]	Food sovereignty strategies during COVID-19 in Colombia	Case study	Mixed-method analysis	Evaluates community and government responses	Highlights resilience and local action	Limited generalizability	Comparative studies across Latin America
Chavarro & Mosquera-Becerra (2023) [31]	Food risks during lockdown and sovereignty	Critical review	Pandemic impact assessment/ Qualitative	Captures vulnerability dynamics	Frames hunger as systemic risk	Temporality limits scope	Design emergency food systems with a sovereignty focus
DeJesus et al. (2024) [22]	Social connections and food security in uncontrolled diabetes	Population-based study	Survey and statistical modeling/ Quantitative	Links social isolation to poor outcomes	Emphasizes social determinants	Focused on uncontrolled cases	Promote community-based support systems
Gomes et al. (2024) [25]	Food insecurity and adolescent health during COVID-19	Epidemiological study	Quantitative analysis	Highlights youth vulnerability in crises	Links food insecurity to health risk behaviors	Focused on the pandemic period	Expand longitudinal studies on youth nutrition
Gomez et al. (2023) [21]	Food security during pregnancy and diabetes	Epidemiological study	Perinatal health analysis/ Quantitative	Highlights maternal vulnerability	Advocates prenatal nutrition support	Focused on pregnancy	Extend to postpartum and child health
Gonzalez et al. (2025) [33]	Food as medicine through justice and sovereignty lenses	Review	Theoretical exploration/Mixed Methods	Integrates food access with clinical care	Promotes justice-based health models	Conceptual without policy detail	Advance clinical-community food interventions
Gu et al. (2024) [16]	Diet quality and glycemia over time	Longitudinal study	Medicaid population tracking/ Quantitative	Tracks food insecurity’s long-term effects	Connects diet shifts to glycemic control	Focused on prediabetes	Expand to broader chronic care models
Gucciardi et al. (2014) [39]	Review of food insecurity and diabetes intersection	Systematic literature review	Qualitative synthesis	Maps, mechanisms, and social determinants	Identifies research gaps and policy blind spots	Data may be outdated; limited cultural diversity	Recommends culturally sensitive and longitudinal studies
Hasan-Ghomi et al. (2015) [8]	Food security and diabetes risk in Tehran	Cross-sectional study	Population-based survey/Quantitative	Highlights urban food insecurity	Connects diet quality to diabetes risk	Older data set	Update with current urban trends
Kennedy (2024) [1]	Role of community nurses in food security and diabetes care	Commentary	Narrative perspective/ Qualitative	Highlights frontline care in community settings	Urges stronger nursing roles in chronic care	Limited empirical data	Expand nursing education and community outreach
Levi et al. (2023) [37]	Intersections between food insecurity and diabetes	Narrative overview	Qualitative overview	Proposes integrated clinical and public health strategies	Highlights systemic barriers and dual-impact interventions	Limited empirical testing; mostly conceptual	Encourages dual-purpose interventions and longitudinal studies
Li et al. (2022) [3]	Gestational diabetes and food insecurity	Epidemiological study	NHANES data analysis/ Quantitative	Connects maternal health to chronic disease risk	Highlights intergenerational impact	Cross-sectional design	Longitudinal studies on maternal nutrition
Lofton et al. (2023) [30]	Food environment interventions using the sovereignty framework	Systematic review	Evidence synthesis/Mixed methods	Links interventions to diet-related outcomes	Uses sovereignty as an evaluative lens	Heterogeneity of studies	Tailor food environments to cultural contexts
Massey et al. (2024) [6]	Glycemic goals and food insecurity in Medicare patients	Survey study	Claims and survey data/Quantitative	Informs elderly diabetes care policy	Connects food access to treatment outcomes	Focused on U.S. Medicare	Adapt for aging populations in emerging countries
Maudrie et al. (2023) [27]	The distinction between food security and sovereignty	Review	Conceptual clarification/Qualitative	Frames sovereignty as empowerment	Differentiates survival vs. thriving	Limited policy examples	Apply the sovereignty lens to public health interventions
Michels et al. (2022) [13]	Mental health, food insecurity, and diabetes	Survey study	Self-reported data/ Quantitative	Highlights psychosocial dimensions	Advocates for holistic care models	Focused on severe mental illness	Integrate mental health into diabetes care
Minari et al. (2024) [9]	Nutritional intervention and glycemic control	Intervention clinical study	Non-randomized intervention study/ Quantitative	High – informs dietary policy and clinical guidelines	Strong evidence of impact on biomarkers	Limited generalizability beyond the study population	Suggests scaling interventions in LMICs
Mosquera & Osorio (2025) [32]	Political and ethical dimensions of hunger and sovereignty	Editorial/ Call to action	Commentary/Qualitative	Raises governance and equity questions	Frames hunger as a public health failure	No empirical data	Advocate for policy reforms grounded in sovereignty
Namkhah et al. (2023) [17]	AI in sustainable food and nutrition systems	Review	Narrative review/ Qualitative	Moderate – highlights tech integration in food systems	Broad overview of AI applications	Lacks empirical validation	Encourages AI-driven policy frameworks
Nayak et al. (2023) [18]	AI for insulin management in T2D	Randomized clinical trial	Randomized clinical trial/Quantitative	High potential for digital health integration	Demonstrates feasibility and efficacy	Focused on tech-accessible populations	Recommends expansion to underserved settings
Osborn et al. (2023) [2]	Neighborhood cohesion as a protective factor	Observational study	Quantitative analysis	Links social cohesion to diabetes outcomes	Emphasizes community resilience	Focused on the Latino population	Apply findings to broader ethnic groups
Reid et al. (2022) [11]	Diabetic ketoacidosis and food insecurity	Cross-sectional study	Clinical data analysis/ Quantitative	Links food insecurity to acute complications	Differentiates between types of outcomes	Focused on youth	Extend to adult populations
Ruelle et al. (2022) [34]	Indigenous knowledge, food sovereignty, and climate adaptation	Case study	Ecological and cultural analysis/ Quantitative	Links sovereignty to environmental resilience	Highlights traditional knowledge systems	Region-specific insights	Incorporate ecological calendars into food policy planning
Shaheen et al. (2021) [20]	Dietary quality and glycemic control	Observational study	Clinical nutrition data/Quantitative	Links food quality to diabetes outcomes	Supports dietary interventions	Limited geographic diversity	Tailor nutrition programs to local contexts
Sonko et al. (2023) [19]	AI prediction models for T2D	Scoping review	Strategic and theoretical review/ Qualitative	Moderate—supports early detection strategies	Synthesizes predictive modeling approaches	Limited depth in policy translation	Calls for real-world validation and an equity lens
Soto et al. (2023) [24]	Agricultural complementarity and food sovereignty in Latin America	Review	Strategic and theoretical review/ Qualitative	Supports regional food self-sufficiency	Advocates for cooperation across countries	Limited country-specific data	Strengthen regional policy articulation
Strings et al. (2016) [15]	Race and sex differences in food insecurity and diabetes	Epidemiological study	Stratified analysis/ Quantitative	Reveals intersectional disparities	Urges equity-focused policies	U.S.-centric	Apply intersectionality in global contexts
Tezera et al. (2022) [4]	Dietary adherence and food security in Ethiopia	Cross-sectional study	Hospital-based survey/ Quantitative	Reveals gaps in counseling effectiveness	Links food insecurity to poor adherence	Limited geographic scope	Strengthen nutrition counseling in low-resource settings
Thomas et al. (2021) [7]	Food insecurity and chronic disease comorbidities	Review	Literature synthesis/ Qualitative	Frames food insecurity as a multisystemic issue	Links diabetes to mental health and CVD	Broad scope, lacks specificity	Develop integrated care models
Tran et al. (2023) [5]	COVID-19 impact on glycemic control and food security	Cross-sectional study	Pandemic analysis/ Quantitative	Shows disparities in diabetes care	Highlights vulnerability during crises	Temporally limited	Build emergency food-health systems
United Nations (2025) [26]	Sustainable Development Goals and global food justice	International Agreement	Global framework/Mixed methods	Aligns national goals with SDGs	Promotes integrated development strategies	Non-prescriptive and general	Localize SDG targets in national food policies
Whitehouse et al. (2024) [35]	Food insecurity interventions for diabetes and prediabetes	Systematic review	RE-AIM framework synthesis/ Quantitative	Evaluates the scalability and impact of multisectoral programs	Highlights gaps in implementation and equity	Limited focus on emerging countries	Apply RE-AIM to intersectoral strategies in LMICs
Wylie-Rosett & DiMeglio (2023) [38]	Strategies to reduce food insecurity in diabetic populations	Policy-focused discussion/ Call to action	Qualitative	Advocates for screening and policy reform in diabetes care	Urges multisectoral collaboration and urgency	No primary data; relies on existing frameworks	Suggests implementation of research and evaluation metrics
York et al. (2020) [14]	Prescriptions for vegetables and health outcomes	Pilot study	Intervention-based/ Quantitative	Promotes food-as-medicine approach	Shows promise in improving food security	Small sample size	Scale up community-based interventions

AI (Artificial intelligence); CVD (Cardiovascular disease); LMICs (Low- and middle-income countries); NHANES (National health and nutrition examination survey); SDGs (Sustainable development goals); SNAP (Supplemental nutrition assistance program); T2D (Type 2 diabetes); UN; RE-AIM (Reach, effectiveness, adoption, implementation, maintenance).

## Data Availability

No new data were created or analyzed in this study. Not applicable.

## Abbreviations

The following abbreviations are used in this manuscript:

Abbreviation	Meaning
AI	Artificial intelligence
CBHI	Community-Based Health Insurance
COVID-19	Coronavirus disease-19
CVD	Cardiovascular disease
FAP	Food acquisition program
LMICs	Low- and middle-income countries
NHANES	National health and nutrition examination survey
PMID	PubMed identifier
PNAE	National school feeding program—Brazil
RCT	Randomized controlled trial
RE-AIM	Reach, effectiveness, adoption, implementation, maintenance
SDGs	Sustainable development goals
SNAP	Supplemental Nutrition Assistance Program
T2D	Type 2 diabetes
UN	United nations
UNIFESP	Federal University of São Paulo
WHO	World Health Organization
